# Systematic literature review on early clinical evidence for immune-resolution therapies and potential benefits to patients and healthcare providers

**DOI:** 10.3389/fimmu.2024.1425478

**Published:** 2024-10-17

**Authors:** Paul Klekotka, Louis Lavoie, Beth Mitchell, Ike Iheanacho, Russel Burge, Andrea Cohee, Joanne Puckett, Ajay Nirula

**Affiliations:** ^1^ Eli Lilly and Company, Indianapolis, IN, United States; ^2^ Evidera Inc, Montreal, QC, Canada; ^3^ Evidera Inc, London, United Kingdom; ^4^ Division of Pharmaceutical Sciences, Winkle College of Pharmacy, University of Cincinnati, Cincinnati, OH, United States

**Keywords:** asthma, atopic dermatitis, immune resolution, rheumatoid arthritis, SLE - systemic lupus erythematosus, ulcerative colitis

## Abstract

**Introduction:**

Several current therapies for autoimmune diseases do not provide sustained remission. Therapies that focus on the restoration of homeostasis within the immune system (i.e., immune resolution) could overcome the limitations of current therapies and provide more durable remission. However, there is no established consensus on appropriate clinical trial designs and endpoints to evaluate such therapies. Therefore, we conducted a systematic literature review (SLR) focusing on five index diseases (asthma, atopic dermatitis, rheumatoid arthritis, systemic lupus erythematosus [SLE], and ulcerative colitis) to explore published literature on 1) expert opinion on immune-resolution outcomes that should be measured in clinical trials; and 2) quantification of immune resolution in previous clinical trials.

**Methods:**

The SLR was conducted in accordance with the Preferred Reporting Items for Systematic Reviews and Meta-Analyses (PRISMA) guidelines. Embase and MEDLINE databases were systematically searched (2013–2023) for published English language articles. Conference proceedings (2020–2022) from American Academy of Dermatology, American College of Rheumatology, Digestive Disease Week, European Alliance of Associations for Rheumatology, and European Academy of Dermatology and Venereology were searched to include relevant abstracts. The study protocol was registered in PROSPERO (CRD42023406489).

**Results:**

The SLR included 26 publications on 20 trials and 12 expert opinions. Expert opinions generally lacked specific recommendations on the assessment of immune resolution in clinical trials and instead suggested targets or biomarkers for future therapies. The targets included thymic stromal lymphopoietin (*TSLP*) in asthma; T helper (Th)2 and Th22 cells and their respective cytokines (interleukin [IL]-4R and IL-22) in atopic dermatitis; inhibitory/regulatory molecules involved in T-cell modulation, and protein tyrosine phosphatase, non-receptor type 22 (*PTPN22*) in rheumatoid arthritis; low-dose IL-2 therapy in SLE; and pro-resolution mediators in ulcerative colitis and asthma. In the interventional studies, direct biomarker assessments of immune resolution were the number/proportion of regulatory T-cells (Treg) and the ratio Th17/Treg in SLE and rheumatoid arthritis; the number of T follicular helper cells (Tfh), Th1, Th2, Th17, and Th22 in atopic dermatitis, rheumatoid arthritis, and SLE; and mucosal proinflammatory gene signatures (tumor necrosis factor [*TNF*], interleukin 1 alpha [*IL1A*], regenerating family member 1 alpha [*REG1A*], *IL8*, interleukin 1 beta [*IL1B*], and leukocyte immunoglobulin-like receptors A [*LILRA*]) in ulcerative colitis. Several studies reported a statistically significant relationship between clinical remission and immune-resolution biomarkers, suggesting a link between T-cell homeostasis, cytokine production, and disease activity in autoimmune diseases.

**Discussion:**

Existing literature does not offer clear guidance on the evaluation of immune resolution in interventional studies. Further research and consensus are needed to assess a treatment’s ability to induce long-term remission or low disease activity.

**Systematic review registration:**

https://www.crd.york.ac.uk/prospero/display_record.php?ID=CRD42023406489, identifier CRD42023406489.

## 1 Introduction

Disruption in the balance between immune activation and self-tolerance may lead to the development of autoimmune diseases (1). Historic therapies for autoimmune diseases are broadly acting and non-specific (e.g., corticosteroids, cytotoxic agents) and may be associated with significant side effects or other safety issues. Current therapies interfere with the activity of key proinflammatory cytokines (e.g., tumor necrosis factor [TNF] inhibitors in inflammatory bowel disease [IBD], and TNF and interleukin [IL]-6 inhibitors in rheumatoid arthritis) or target specific immune cells (e.g., B-cell modulation by belimumab in systemic lupus erythematosus [SLE]) (1, 2). While such treatments can benefit patients, they are associated with adverse events, often fail to provide long-term disease remission, and rarely restore the balance within the immune system (2–4).

Restoration of homeostasis within the immune system in chronic autoimmune diseases is usually referred to as immune resolution (5, 6). Therapies that effectively target this restoration phase of immune response (e.g., by acting through immune checkpoint inhibitory receptors or regulatory T-cells [Treg]) could represent a fundamental shift in disease management and help overcome the limitations of current treatments. Specifically, these therapies could provide long-term low disease activity or even remission, with a reduced dependency on corticosteroids or other immunosuppressants (7). Sustained remission with longer treatment intervals or potential for drug withdrawal could ultimately help achieve curative efficacy and improved safety.

There is no established consensus on appropriate clinical trial designs and endpoints to evaluate therapies targeting immune resolution. Therefore, we conducted a systematic literature review (SLR) to explore published literature on 1) expert opinion on immune-resolution outcomes that should be measured in clinical trials; and 2) quantification of immune resolution in previous clinical trials. The SLR focused on five index diseases (asthma, atopic dermatitis, rheumatoid arthritis, SLE, and ulcerative colitis) in which disturbance of immune homeostasis may have a pathogenic role and no approved therapies are available that target immune resolution.

## 2 Methods

### 2.1 Study design

An exploratory SLR was conducted to identify clinical trials and expert opinions on the therapeutic value of immune-resolution therapies in five index diseases: rheumatoid arthritis, asthma, atopic dermatitis, ulcerative colitis, and SLE. The SLR was conducted in accordance with the Preferred Reporting Items for Systematic Reviews and Meta-Analyses (PRISMA) guidelines (8). The study protocol was registered in PROSPERO (CRD42023406489).

### 2.2 Data sources and search strategy

Comprehensive literature searches were conducted using Embase and MEDLINE electronic databases (via OvidSP platform) for articles published in the English language between January 1, 2013 and February 22, 2023. Searches were conducted using a combination of free-text search terms and controlled-vocabulary terms specific to each database as recommended by the Cochrane Collaboration (9). The detailed search strategy is provided in **Supplementary Table 1** (Embase) and **Supplementary Table 2** (MEDLINE). In addition, conference proceedings from the American Academy of Dermatology, American College of Rheumatology, Digestive Disease Week, and European Alliance of Associations for Rheumatology (indexed in Embase) and European Academy of Dermatology and Venereology (searched manually) held between 2020 and 2022 were searched to identify relevant abstracts. The searches were restricted to the index diseases and were based on separate search terms for ‘immune’, ‘autoimmune’, ‘inflammation’, ‘resolution phase’, ‘immunometabolism’, ‘immunoregulator’, ‘checkpoint inhibit$’, ‘Tregs’, and for potential target molecules of immune resolution (e.g., IL-2 conjugates, programmed cell death protein-1 [PD-1] agonists, CD200 receptor [CD200R] agonists, B- and T-lymphocyte attenuator [*BTLA*] agonists).

### 2.3 Eligibility criteria

The eligibility of studies was based on the pre-defined population, interventions and comparators, outcomes, and study design (PICOS) criteria (**Table 1**). Interventional trials and expert opinions/expert opinion-driven reviews on immune-resolution outcomes in adult patients with a confirmed diagnosis of asthma, atopic dermatitis, rheumatoid arthritis, ulcerative colitis, or SLE were included. Furthermore, articles reporting any approved or investigational therapy that would be (or has the potential to be) considered an immune-resolution therapy were included.

**Table 1:** PICOS criteria.

Domain	Eligible	Ineligible
**Population**	Adult patients with a confirmed diagnosis of one of the following immunology diseases: • Asthma • Atopic dermatitis • Rheumatoid arthritis • Systemic lupus erythematosus • Ulcerative colitis	• Patients without a confirmed diagnosis of one of the immunology diseases of interest • Children (aged ≤17 years)
**Intervention**	Any approved or investigational therapy that would be considered (or has the potential to be considered) an immune-resolution therapy including PD-1 agonists, IL-2 conjugates, CD200R agonists, Treg modulators, and *BTLA* agonists	• Surgical interventions • Alternative medicine
**Comparators**	Any or none	NA
**Outcomes**	• Any outcome relating to immune resolution • Expert opinion on immune-resolution outcomes expected in the future	Surrogate endpoints not definitively linked to immune resolution
**Study design**	• Interventional trials • Expert opinions	Reviews reporting secondary data
**Time frame**	2013-present	Publications before 2013
**Language**	English	Non-English

*BTLA*, B- and T-lymphocyte attenuator; CD200R, CD200 receptor; IL-2, interleukin-2; NA, not applicable; PD-1, programmed cell death protein-1; Treg, regulatory T-cells.

Articles reporting surrogate endpoints not definitively linked to immune resolution, reviews reporting secondary data, and duplicate and non-English language articles were excluded.

### 2.4 Study selection and data extraction

The titles, abstracts, and full text of articles were exported into Distiller Systematic Review software (DistillerSR; Evidence Partners, Ottawa, Ontario, Canada) for screening. Articles were screened by one single reviewer (PK or ZEG), and a second reviewer (II) conducted a 25% random screening of excluded publications for quality assurance. Any discrepancies were resolved by mutual consensus or by involving a third team member (LL) to reach a final decision.

Data extraction was performed using a standardized data extraction form and after consensus on data extraction guidelines. All data were extracted by one reviewer (PK or ZEG) and then validated by a second reviewer (LL). A third reviewer (II) was consulted to resolve any disagreements. Data extractors or validators were not blinded to any study information.

For added quality assurance, a final check was completed once all information was extracted to ensure consistency in the reporting of information across publications.

### 2.5 Outcomes

Immune-resolution outcomes, including laboratory and clinical measures, and expert opinions on these outcomes that could be relevant for clinical trials of the index diseases were included.

While treatment efficacy was not a primary focus of the review, these data were collected to provide additional perspective on the studies and how they had been conducted.

### 2.6 Study risk-of-bias assessment

A formal risk-of-bias assessment was not performed for this review because the research aimed to seek published opinions and outcomes selected for use in individual trials, which could automatically be considered as being ‘biased’. Such labeling might offer an unhelpful or even misleading perspective of the identified publications about what the research was attempting to explore. Study quality and relevance were, however, considered in terms of study design, sample size, and generalizability.

### 2.7 Synthesis of results

Findings were narratively synthesized. Studies were grouped according to the key themes identified to allow connections with the review objectives to be analyzed and summarized. All included interventional trials were assessed for quality based on sample size and whether the trial was protocol driven.

## 3 Results

### 3.1 Study selection

**Figure 1:**
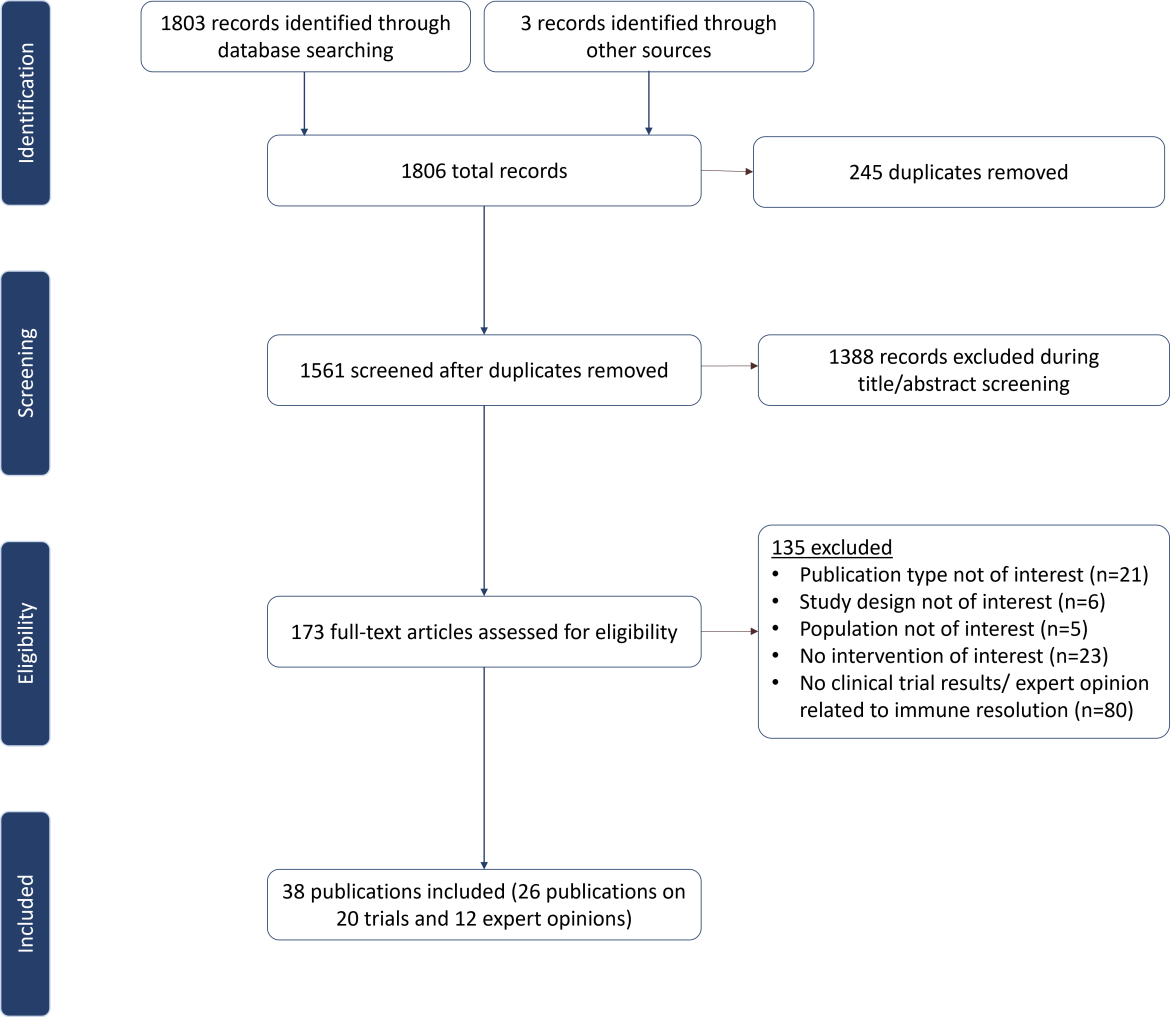
PRISMA diagram.

The indexed database searches yielded 1,558 unique articles, 35 of which met the study inclusion criteria (**Figure 1**). An additional three articles were identified through supplementary searches, resulting in 38 included publications, i.e., 26 publications on 20 trials and 12 expert opinions (**Figure 1**).

### 3.2 Study characteristics

#### 3.2.1 Expert opinions

Overall characteristics of included expert opinions (n=2, asthma; n=2, atopic dermatitis; n=1, rheumatoid arthritis and SLE; n=2, SLE; n=2, ulcerative colitis [and IBD]; n=1, ulcerative colitis and asthma; n=2, rheumatoid arthritis) are summarized in **Table 2**. All expert opinions were from multiple specialists/commentators, and authors from university/hospital institutions, but no consensus documents or publications from academic bodies were identified.

**Table 2:** Expert opinion characteristics and recommendations^a^.

Author, year	Institution/affiliation	Funding/Sponsor	Immune-resolution outcomes proposed/recommended	Authors’ conclusions
Asthma				
**Menzies-Gow, 2021 (** 16)	Royal Brompton Hospital, Respiratory Medicine. The Breathing Institute, Children’s Hospital Colorado. Department of Pediatrics, University of Colorado School of Medicine, Anschutz Medical Campus. Department of Medicine, Allergy, Pulmonary and Critical Care Medicine, University of Wisconsin-Madison.	None	None	Biologics can achieve some but not all criteria for remission and efforts to develop treatments achieving full remission criteria still need to be sustained.
**Gauvreau, 2020 (** 17)	Department of Medicine, McMaster University. Respiratory & Immunology, BioPharmaceuticals Medical. Translational Science and Experimental Medicine, Research and Early Development, Respiratory & Immunology, BioPharmaceuticals R&D.	AstraZeneca and Amgen	Thymic stromal lymphopoietin (*TSLP*) expression and biomarkers for patients who respond to *TSLP* therapy.	*TSLP* blockade could serve as an immunomodulatory function in asthma, restoring homeostatic balance.
Atopic Dermatitis				
**Kalamaha, 2019 (** 41)	Department of Internal Medicine, University of North Dakota School of Medicine and Health Sciences. Hematology and Medical Oncology, Sanford Health. Department of Pediatrics, University of North Dakota School of Medicine and Health Sciences. Allergy and Immunology, Sanford Health.	None	None. The authors stated that additional research was needed to better understand the pathogenesis of atopic dermatitis, and suggested that future research will likely focus on the modulation or inhibition of certain cytokines, particularly IL-4, IL-13, IL-17, IL-31, and JAK-signal transducer and activator of transcription (STAT) inhibition.	Several trials on the use of biologics for AD did not demonstrate long-term safety and efficacy. Future research should be directed to develop biomarkers for different AD phenotypes to allow for targeted therapy of AD.
**Guttman-Yassky, 2013 (** 18)	National Jewish Health, Department of Pediatrics, Colorado.	National Institutes of Health grants R01 AR41256	Targeting T helper (Th)2 or Th22 (and the corresponding cytokines IL-4R and IL-22, respectively) simultaneously or sequentially might be needed to maximize effectiveness.	Validated biomarkers for disease improvement in AD are available and should be used to determine whether the clinical resolution of the disease is also accompanied by molecular and tissue resolution. This should help for the development of biologic therapies directed at pathways driving AD.
Rheumatoid Arthritis				
**Hemmatzadeh, 2022 (** 19)	Tabriz University of Medical Sciences. Immunology Research Center, Tabriz University of Medical Sciences Department of Immunology, Faculty of Medicine, Tabriz University of Medical Sciences Department of Biology, Faculty of Natural Science, University of Tabriz Non‐Communicable Diseases Research Center, Alborz University of Medical Sciences Department of Immunology, School of Medicine, Alborz University of Medical Sciences Research Center for Integrative Medicine in Aging, Aging Research Institute, Tabriz University of Medical Sciences	Tabriz University of Medical Sciences	Potential markers/outcomes related to inhibitory and regulatory pathways, such as *CTLA‐4*, PD‐1/PD‐L1, *BTLA*, *LAG3*, TIM3, *TIGIT*, and *VISTA*.	Downmodulation of the molecules involved in the inhibitory and regulatory pathways, such as *CTLA‐4*, PD‐1/PD‐L1, *BTLA*, *LAG3*, TIM3, *TIGIT*, and *VISTA* by specific antibodies or recombinant proteins should be used in the future for controlling diseases mediated by disturbed T cell‐associated immune responses such as those seen in RA.
**Carmona, 2018 (** 20)	Departamento de Genética e Instituto de Biotecnología, Universidad de Granada. Instituto de Parasitologia y Biomedicina Lopez-Neyra, Consejo Superior de Investigaciones Cientificas	Ramón y Cajal’ programme of the Spanish Ministry of Economy and Competitiveness (RYC-2014-16458); Instituto de Salud Carlos III (ISCIII), Spain, through the RETICS Program RD16/0012/0004 (RIER); and the EU/EFPIA Innovative Medicines Initiative Joint Undertaking PRECISESADS	*PTPN22*-encoded expression of lymphoid-specific tyrosine phosphatase (LYP)	*PTPN22* encodes a LYP which is a master regulator of the immune response. Understanding and controlling the pathogenic implications of the *PTPN22* risk alleles may help to achieve a complete remission of RA or at least to slow progression.
SLE				
**Akbarzadeh, 2023 (** 21)	Department of Rheumatology and Clinical Immunology, University of Lubeck	German Research Foundation (EXC2167)	Treg recovery and expansion maintained by IL-2	Low-dose IL-2 in combination with other immunotherapeutics including biologics provides synergistic and complementary immunomodulatory effects in SLE.
**Nakayamada, 2022 (** 42)	The First Department of Internal Medicine, School of Medicine, University of Occupational and Environmental Health	Ministry of Health, Labor and Welfare of Japan, the Ministry of Education, Culture, Sports, Science and Technology of Japan (#JP20K08815, JSPS KAKENHI), and University of Occupational and Environmental Health (UOEH), Japan, through UOEH Grant for Advanced Research	Activation of innate immunity pathways stimulates the release of the cytokines BAFF, type I interferon, type II interferon, IL-12, and/or IL-23 and these activate acquired immunity pathways including T cell differentiation and activation, B-cell class switching, and differentiation into antibody-producing cells. Also, serum levels of soluble BAFF and IFN-α rise with increased disease activity.	The pathogenesis of SLE involves abnormalities in both acquired and innate immune systems linked to the action of various cytokines, and in turn to JAKs. This makes JAK inhibition a key potential treatment target in SLE.
Rheumatoid Arthritis and SLE				
**Iwata, 2021 (** 22)	The First Department of Internal Medicine, University of Occupational and Environmental Health, School of Medicine, Kitakyushu.	JSPS (Japan Society for the Promotion of Science) grant number #JP16K09928	None. The authors considered various strands of evidence on mechanisms of metabolic regulation of B cells and other immune cells as potential treatment targets in rheumatoid arthritis and SLE but concluded that more research is needed on this area, without proposing specific measures by how immunoregulatory effects might best be assessed.	Although drugs that target mTOR, AMPK, and glycolytic systems such as sirolimus, rapamycin, and metformin have shown some efficacy and tolerability in clinical trials in patients with SLE, they have not led to major developments in therapeutic approaches. A better understanding of intrinsic immunometabolism mechanisms including those for B cells and other immune cells, is needed, and may aid the development of novel treatments for SLE.
Ulcerative Colitis and Asthma				
**Perucci, 2017 (** 23)	Departamento de Análises Clínicas e Toxicológicas, Faculdade de Farmácia, Universidade Federal de Minas Gerais. Programa de Pós-Graduação em Análises Clínicas e Toxicológicas, Universidade Federal de Minas Gerais. Programa de Pós-Graduação em Ciências Farmacêuticas, Universidade Federal de Minas Gerais. Departamento de Bioquímica e Imunologia, Instituto de Ciências Biológicas, Universidade Federal de Minas Gerais	Conselho Nacional de Desenvolvimento Científico e Tecnológico CNPq (447452/2014-2) and Fundação de Amparo à Pesquisa do Estado de Minas Gerais FAPEMIG (APQ-03318-15) fellowship	Markers of pro-resolving mechanisms, such as those involving alterations in the levels or function of mediators annexin A1 (*ANXA1*) and specialized pro-resolving lipid mediators (SPMs; e.g., arachidonic acid, n-6 PUFA)	The complex interplay between pro-resolving mediators such as *ANXA1* and SPMs highlights the central role of immune resolution in tissue homeostasis. Evidence in asthma suggests that deficiencies of *ANXA1* and LXA4 (an SPM) could occur early in disease progression. Also, *ANXA1* and SPM-based interventions might represent novel therapeutic approaches for patients with asthma. Evidence in ulcerative colitis has suggested that there is deficient synthesis of LXA4 and that (as with *ANXA1*), LXA4 expression is increased in clinical remission. Such data indicate that SPM analogs might be potential candidate treatments for IBD.
Ulcerative Colitis				
**Porter, 2018 (** 43)	School of Medicine, Medical Sciences and Nutrition, University of Aberdeen. Laboratory of Molecular Immunoregulation, Cancer and Inflammation Program, National Cancer Institute, National Institutes of Health	NR (details provided only of support received by some of the authors but not specifically for the reported study)	None. The authors discussed the relevance of B cells and Treg.	There is no universally effective treatment for IBD and current interventions do not induce lasting remission in all, and/or are associated with long-term adverse effects, which may be chronic. A personalized medicine approach is needed given the significant variability between patients with regard to disease activity, progression over time, and response to treatment.
**Leiman, 2014 (** 44)	Division of Gastroenterology, Perelman School of Medicine, University of Pennsylvania.	NR	None. The authors highlighted targeting cellular adhesion (e.g., through vedolizumab) and inflammatory cell signaling as key strategies for the management of ulcerative colitis.	There is potential for new and effective therapies for patients with IBD. Vedolizumab is one such novel therapy for ulcerative colitis.

a All expert opinions were review articles and were from multiple specialists/commentators.

AD, atopic dermatitis; AMPK, adenosine-monophosphate-activated protein kinase; BAFF, B-cell–activating factor; *BTLA*, B-cell lymphocyte attenuator; *CTLA-4,* cytotoxic T-lymphocyte–associated antigen-4; IBD, inflammatory bowel disease; IFN, interferon; IL, interleukin; JAK, Janus kinase; *LAG3,* lymphocyte activation gene 3; mTOR, mammalian target of rapamycin; NR, not reported; PD-1, programmed cell death 1; PD-L1, programmed cell death-ligand 1; *PTPN22,* protein tyrosine phosphatase, non-receptor type 22; PUFA, polyunsaturated fatty acids; RA, rheumatoid arthritis; SLE, systemic lupus erythematosus; *TIGIT,* T cell immunoreceptor with immunoglobulin and immunoreceptor tyrosine-based inhibitory motif domains; TIM3, T cell immunoglobulin and mucin domain 3; Treg, regulatory T-cells; *VISTA,* V-domain immunoglobulin suppressor of T cell activation.

#### 3.2.2 Interventional studies

Overall characteristics of included interventional studies (n=9, SLE; n=7, rheumatoid arthritis; n=3, atopic dermatitis; n=1, ulcerative colitis; n=0, asthma) are summarized in **Supplementary Table 3**. Of the 20 studies included, 9 were phase II studies, 7 were randomized controlled trials, 2 were phase I or phase I/II studies, and 2 were interventional prospective studies. Across studies, the sample size ranged from 16 (10) to 321 (11) patients. The mean age across the different study groups ranged from 29.8 (12) to 56.4 (13) years, and the proportion of females ranged from 57.7% (14) to 100% (15).

### 3.3 Recommendations/highlights from expert opinions

In general, there was a lack of specific recommendations on ways of assessing immune resolution in clinical trials. Instead, the authors summarized specific pathophysiological evidence suggesting restoration of immune homeostasis. Only a few expert opinions directly recommended outcomes to be potentially used to assess immune resolution in at least one of the five index diseases. Most of the reviews/expert opinions suggested different targets or biomarkers that future therapies should focus on to achieve immune resolution.

#### 3.3.1 Asthma

In a review of the relationship between biologics and remission in asthma, four subtypes of asthma remission were identified: clinical remission – on and off treatment, and complete remission – on and off treatment (16). The authors did not discuss effectiveness beyond remission.

Gauvreau et al. summarized in their review the crucial role of thymic stromal lymphopoietin (*TSLP*), an epithelial cytokine (alarmin), in the pathogenesis of asthma and the therapeutic potential of anti-*TSLP* monoclonal antibodies in asthma (17).

#### 3.3.2 Atopic dermatitis

Guttman-Yassky et al. commented on the potential importance of T helper (Th)2 and Th22 cells and their respective cytokines (IL-4R and IL-22) in the etiology of atopic dermatitis. Treatment strategies focusing on targeting Th2 and Th22 simultaneously or sequentially might help maximize treatment effectiveness (18).

#### 3.3.3 Rheumatoid arthritis

Inhibitory/regulatory molecules, such as cytotoxic‐T‐lymphocyte antigen 4 (*CTLA‐4*), PD‐1/programmed cell death-ligand 1 (*PD‐L1*), lymphocyte activation gene 3 (*LAG3*), T cell immunoglobulin and mucin domain 3 (TIM3), T cell immunoreceptor with immunoglobulin and immunoreceptor tyrosine-based inhibitory motif domains (*TIGIT*), V‐domain immunoglobulin suppressor of T cell activation (*VISTA*), and *BTLA* play a key role in the modulation of the activation and tolerance of T cells in rheumatoid arthritis (19).

The gain-of-function variant of protein tyrosine phosphatase non-receptor type 22 (*PTPN22*) encodes the expression of a lymphoid-specific tyrosine phosphatase (master regulator of the immune response) and increases the risk of rheumatoid arthritis. Thus, *PTPN22* could be a potential therapeutic target for rheumatoid arthritis (20).

#### 3.3.4 SLE

Low-dose IL-2 therapy plays a key role in the proliferation and survival of Treg required to restore homeostatic balance in SLE and is recognized as a potential targeted treatment approach (21).

#### 3.3.5 Rheumatoid arthritis and SLE

In a review on the metabolism of lymphocytes in rheumatoid arthritis and SLE, Iwata and Tanaka acknowledged the relative efficacy of immune-metabolic modulators (e.g., sirolimus/rapamycin, metformin) in clinical trials but argued that further development was needed to elucidate the mechanisms of immunometabolism, especially for B cells (22).

#### 3.3.6 Ulcerative colitis and asthma

Perucci et al. discussed the protective role of pro-resolving mediators such as annexin A1 (*ANXA1*) and specialized pro-resolving lipid mediators (derived from essential fatty acids) in promoting resolution in inflammatory diseases and setting the foundation for a novel therapeutic strategy coined ‘resolution pharmacology’ (23).

### 3.4 Biomarkers used to assess immune-resolution potential in interventional studies

Outcomes used to assess clinical remission in the index diseases are detailed in **Table 3**. Biomarkers used to assess immune-resolution potential are briefly summarized by disease below and full details are provided in **Table 3**.

**Table 3:** Outcomes used to assess clinical remission and potential immune resolution in included trials.

Disease	Clinical Remission Markers	Markers of Potential Immune Resolution
**Atopic Dermatitis**	• IGA 0/1 and EASI-75 (26)	• IL-22 serum levels (24, 25) • T helper (Th)1/Th2/Th17/Th22 gene expression (24) • Markers of general inflammation: MMP12, hyperplasia K16, Th2 immune response (C-C motif chemokine ligand [*CCL*]17, *CCL18*, *CCL26*), and Th17/Th22 immune response (S100 calcium-binding protein A8, A9, and A12 [*S100A8*, *S100A9*, *S100A12*]) *(* 26)
**Rheumatoid Arthritis**	• DAS28 or DAS28-ESR (14, 28, 30–32) • DAS28-ESR<2.6 (11, 13, 29) • DAS28-CRP ≤ 2.6 (33) • CDAI ≤ 2.8 (33) • Reduction in DMARD daily dose (methotrexate, leflunomide, or hydroxychloroquine) (28)	• Treg counts and/or percentage (13, 14, 27–33) • Th17 counts and/or percentage (13, 14, 27–30, 32, 33) • Th17/Tc17 ratio (33) • Th17/Treg ratio (14, 31) • Th1 counts (30, 32) • Tfh counts (32) • CD4 downmodulation (11) • *CD56^bri^* NK cell counts (27), *IL-17A*, *IFN-γ*, and IL-21 levels (27)
**SLE**	• SELENA-SLEDAI ≤2 (38) • Prednisone tapered ≥ 50% (45) • Reduction in prednisone daily dose (34–36, 38) • Imbalanced Tfh and Tfr cell association with disease activity (12)	• Treg counts and/or percentage (12, 15, 34–38) • Th17 counts and/or percentage (35, 36) • Th17/Treg ratio (12, 35, 36) • Tfh counts (37) • Levels of cytokines (IL-2, IL-4, IL-17) (34, 37) • C3-C4 complement levels (34) • Cell counts of different B-cell populations (39)
**Ulcerative Colitis**	• Mayo score ≤2, rectal bleeding score of 0, and endoscopy ≤1 (10)	• Mucosal proinflammatory gene signature (*TNF*, *IL1A*, *REG1A*, *IL8*, *IL1B*, and *LILRA*) (10)

CCL, chemokine ligand; CDAI, Clinical Disease Activity Index; DAS28, Disease Activity Score-28 joints; DAS28-CRP, Disease Activity Score-28 joints-C-reactive protein; DAS28-ESR, Disease Activity Score-28 joints‐erythrocyte sedimentation rate; DMARD, disease-modifying antirheumatic drug; EASI, Eczema Area and Severity Index; IFN, interferon; IGA, Investigator Global Assessment; IL, interleukin; IL1A, interleukin 1 alpha; IL1B, interleukin 1 beta; K, keratin; LILRA, leukocyte immunoglobulin-like receptors A; MMP, matrix metallopeptidase; NK, natural killer; REG1A, regenerating family member 1 alpha; SELENA-SLEDAI, Safety of Estrogens in Lupus Erythematosus National Assessment-Systemic Lupus Erythematosus Disease Activity Index; SLE, systemic lupus erythematosus; Tfh, T follicular helper cells; Tfr, T follicular regulatory cells; TNF, tumor necrosis factor; Treg, regulatory T-cells.

#### 3.4.1 Atopic dermatitis

Potential biomarkers were reported in three studies and included IL-22 serum levels (24, 25), Th1/Th2/Th17/Th22 gene expression (24), and markers of general inflammation (26).

#### 3.4.2 Rheumatoid arthritis

Use of biomarkers Treg counts and/or percentage (13, 14, 27–33); Th17 counts and/or percentage (13, 14, 27–30, 32, 33); Th17/Treg ratio (14, 31); Th1 counts (30, 32); CD4 downmodulation (11); *CD56^bri^* natural killer (NK) cell counts (27); and *IL-17A*, interferon (*IFN*)-*γ*, and IL-21 levels (27) were reported in seven trials.

#### 3.4.3 SLE

Nine studies reported the use of Treg counts and/or percentage (12, 15, 34–38), Th17 counts and/or percentage (35, 36), Th17/Treg ratio (12, 35, 36), serum levels of cytokines (IL-2) (37), C3-C4 complement levels (34), and cell counts of different B-cell populations (39).

#### 3.4.4 Ulcerative colitis

One study reported the use of mucosal proinflammatory gene signatures (*TNF*, interleukin 1 alpha [*IL1A*], regenerating family member 1 alpha [*REG1A*], *IL8*, interleukin 1 beta [*IL1B*], and leukocyte immunoglobulin-like receptors A [*LILRA*]) in patients with ulcerative colitis (10).

### 3.5 Clinical remission outcomes

Clinical remission outcomes as reported by the proportion of patients under a certain threshold of disease activity or with a reduction in the use of standard-of-care drugs (e.g., corticosteroids) are summarized in **Table 3**.

In a phase IIa trial in patients with atopic dermatitis, IL-22 levels were correlated with disease severity measures at baseline: Eczema Area and Severity Index (EASI; p<0.0001) and SCORing Atopic Dermatitis (SCORAD; p=0.001) (25). In a randomized controlled trial (RCT) in patients with rheumatoid arthritis, a negative correlation was observed between baseline CD4 Treg and Disease Activity Score-28 joints (DAS28; r = −0.625, p<0.001) (14). A *post hoc* analysis of an RCT in patients with SLE treated with IL-2 reported a negative correlation between Treg and disease activity as assessed by the erythrocyte sedimentation rate (ESR) (r = −0.382, p<0.01) (12).

### 3.6 Biomarkers used to assess efficacy in trials

The key efficacy results from trials are summarized in **Supplementary Table 3**. Most of the studies reported treatment efficacy as a reduction in the number and/or proportion of effector T-cells or their related gene products, a reduction in the levels of inflammatory cytokines, and/or an increase in the number and/or proportion of Treg cells.

#### 3.6.1 Atopic dermatitis

Amlitelimab, an anti-OX40 ligand (OX40L) monoclonal antibody, decreased IL-22 levels and disease activity (EASI) at 16 weeks (25).

Rocatinlimab (KHK4083), an anti-OX40 monoclonal antibody, and abrocitinib, a Janus kinase (*JAK*)*-1* selective inhibitor, downregulated Th2, Th1, Th17 and Th22-related gene expression (24), and several Th immune response genes (C-C motif chemokine ligand [*CCL*]*17*, *CCL18*, *CCL26*, S100 calcium-binding protein [*S100*]*A8*, *S100A9*, *S100A12*) (26), at 16 and 12 weeks, respectively.

#### 3.6.2 Rheumatoid arthritis

Low-dose IL-2 selectively increased the number of CD4 Treg (14, 27), rebalanced the ratio of Th17/CD4 Treg (14), and decreased *IL-17A* and *IFN-γ* levels (27) in parallel with decreased disease activity (DAS28 and ACR20) (14, 27).

Sirolimus/rapamycin increased (28, 30) or prevented a decrease (29) in CD4 Treg levels with higher rates of complete remission (DAS28 <2.6) versus conventional treatment (29).

#### 3.6.3 SLE

Sirolimus/rapamycin decreased the number of Th17 cells and Th17/Treg ratio (35), increased the number of CD4 Treg, decreased IL-4 and IL-17 levels, and increased C3 and C4 complement levels (34) along with reduction of disease activity (Systemic Lupus Erythematosus Disease Activity Index [SLEDAI]) and use of prednisone (34, 35).

Low-dose IL-2 increased the number of Treg (36, 38), and restored the T follicular regulatory (Tfr)/T follicular helper (Tfh) cell balance (12) in parallel with the reduction of prednisone use (36, 38) and disease activity (Safety of Estrogens in Lupus Erythematosus National Assessment [SELENA]-SLEDAI) (12).

Iberdomide, a high-affinity cereblon ligand, reduced the number of B cells (including those expressing CD268 [TNF receptor superfamily member 13C; *TNFRSF13C*]), and plasmacytoid dendritic cells, and increased the number of Treg and IL-2 levels (37).

#### 3.6.4 Ulcerative colitis

Olamkicept, an inhibitor of IL-6, induced a change of a mucosal proinflammatory gene signature (*TNF*, *IL1A*, *REG1A*, *IL8*, *IL1B*, and *LILRA*) different from the one characterized by remission signatures of anti-TNF (infliximab) or anti-integrin (vedolizumab) in a phase IIa study with relatively low number of patients (N=16, including 9 and 7 patients with ulcerative colitis and Crohn’s disease, respectively) (10).

## 4 Discussion

This SLR explored published literature on current and potential approaches to assess immune resolution in five index diseases (asthma, atopic dermatitis, rheumatoid arthritis, SLE, and ulcerative colitis). Immune resolution represents a new frontier for designing medicines and moving the treatment spectrum closer to cure. Therapies that could lead to clinical remission may be valued by patients, healthcare providers, and population-based decision-makers. However, there is a lack of published systematic collation of expert recommendations and outcomes that could be used to assess the potential benefits of these novel therapies in clinical trials. This SLR has attempted to address this substantial literature gap and the findings presented should improve the understanding of completed and ongoing trials, as well as the design of future interventional studies. In contrast to previously published reviews that considered immune system rebalancing as a target outcome of new treatments for chronic autoimmune diseases (1, 2, 40), we used a rigorous systematic methodology to synthesize the available evidence on immune-resolution outcomes.

A key finding of this review was the lack of expert recommendations/consensus guidelines on immune-resolution outcomes to be assessed in clinical trials. This may be explained by the relatively nascent nature of this therapeutic field and may be challenging for those seeking to design future trials of such therapies. The expert opinions primarily focused on the potential value of future therapies targeting specific immune pathways, including anti-cytokine biologics, immune-metabolic modulators, immune checkpoint molecules, and anti-adhesion molecules.

Most of the interventional studies defined clinical remission as disease activity below a certain threshold or reduction in the use of glucocorticoids/disease-modifying antirheumatic drugs (DMARDs).

More direct biomarker assessments that can be considered as proxies of immune system rebalancing included the number/proportion of Treg cells and the ratio of Th17/Treg in patients with SLE or rheumatoid arthritis, and the number of Tfh, Th1, Th2, Th17, and Th22 cells in atopic dermatitis, rheumatoid arthritis, and SLE. These assessments could be integrated with the existing measures of disease activity and remission.

Furthermore, several studies identified a statistically significant relationship between clinical remission and immune-resolution biomarkers [e.g., imbalanced Tfh/Treg ratio associated with high disease activity [SELENA-SLEDAI] in SLE (12), IL-22 levels correlated with disease activity [EASI and SCORAD] in atopic dermatitis (25), and baseline CD4 Treg negatively correlated with disease activity [DAS28] in rheumatoid arthritis (14)]. Thus, in each case, there was a demonstrable link between T-cell homeostasis, cytokine production, and disease activity in autoimmune disease.

Discussion on treatment targets by experts was in line with the recent trials included in this review. Key examples included targets related to Treg survival and expansion in SLE, and outcomes targeting Th2 and/or Th22 cells in atopic dermatitis. More speculative therapeutic targets such as *PTPN22* in rheumatoid arthritis were also suggested to restore immune homeostasis (20).

The SLR had several limitations. The review was limited to five specific diseases and may not be generalizable to other autoimmune diseases. However, the selected conditions collectively constitute a substantive proportion of autoimmune diseases, and each has high unmet needs despite the use of the current standard of care. As atopic dermatitis and asthma are heterogeneous diseases, possessing both intrinsic (potentially autoimmune) and extrinsic (potentially allergic) phenotypes, these diseases were included in the SLR. Most of the identified trials were early phase II studies, and it is uncertain if subsequent studies will follow the same methodological approaches. The primary objectives and overall conclusions of the individual studies were not specific to the focus of the SLR. However, neither of these factors invalidate our review since these studies are indicators of current thinking and future treatment practices that may provide immune resolution. Expert opinion on this concept was limited and some of the available articles may be outdated since they were published up to 10 years ago. In addition, most of the authors provided less definitive opinions on evidence indicating that therapies may be restoring immune homeostasis.

Clear correlations between the proxy outcomes reported in these studies and accompanying clinical remission/persistently low disease activity are still needed to be widely demonstrated. With improved understanding of immune regulation, it will be important to recognize targeting immune resolution as a treatment strategy that can be beneficially integrated with current therapies.

## 5 Implications

The outcomes found in this review suggest an interplay between the inflammatory process and immune regulation. Currently available published expert opinion and clinical trial data fall short of offering clear guidance on how the potential of new therapies rebalancing the immune system might be identified and quantified in interventional studies. Future research and consensus are needed by incorporating perspectives from patients, clinicians, regulators, and population-based decision-makers on their assessment of a treatment’s value in inducing immune resolution.

## Data Availability

The original contributions presented in the study are included in the article/**Supplementary Material**. Further inquiries can be directed to the corresponding author.

## Glossary

ANXA1: Annexin A1
BAFF: B-cell–activating factor
BTLA: B- and T-lymphocyte attenuator
CCL: C-C motif chemokine ligand
CDAI: Clinical Disease Activity Index
CD200R: CD200 receptor
CTLA-4: Cytotoxic‐T‐lymphocyte antigen 4
DAS28: Disease Activity Score-28 joints
DMARD: Disease-modifying antirheumatic drugs
EASI: Eczema Area and Severity Index
ESR: Erythrocyte sedimentation rate
IBD: Inflammatory bowel disease
IFN: Interferon
IL: Interleukin
IL1A: Interleukin 1 alpha
IL1B: Interleukin 1 beta
JAK: Janus kinase
LAG3: Lymphocyte activation gene 3
LILRA: Leukocyte immunoglobulin-like receptors A
NK: Natural killer
OX40L: OX40 ligand
PD-1: Programmed cell death protein-1
PD-L1: programmed cell death-ligand 1
PICOS: Population, interventions and comparators, outcomes, and study
PRISMA: Preferred Reporting Items for Systematic Reviews and Meta-Analyses
PTPN22: Protein tyrosine phosphatase, non-receptor type 22
RCT: Randomized controlled trial
REG1A: Regenerating family member 1 alpha
S100: S100 calcium-binding protein
SCORAD: SCORing Atopic Dermatitis
SELENA: Safety of Estrogens in Lupus Erythematosus National Assessment
SLE: Systemic lupus erythematosus
SLEDAI: Systemic Lupus Erythematosus Disease Activity Index
SLR: Systematic literature review
SPM: Specialized pro-resolving lipid mediators
STAT: Signal transducer and activator of transcription
Tfh: T follicular helper
Tfr: T follicular regulatory
Th: T helper
TIGIT: T cell immunoreceptor with immunoglobulin and immunoreceptor tyrosine-based inhibitory motif domains
TIM3: T cell immunoglobulin and mucin domain 3
TNF: Tumor necrosis factor
Treg: Regulatory T-cells
TSLP: Thymic stromal lymphopoietin
VISTA: V‐domain immunoglobulin suppressor of T cell activation
