# Supplementary material for: Systematic literature review on early clinical evidence for immune-resolution therapies and potential benefits to patients and healthcare providers
Source: Front Immunol. 2024 Oct 17;15:1425478. doi: 10.3389/fimmu.2024.1425478 (PMC11524942; doi:10.3389/fimmu.2024.1425478)
Supplement: Supplementary file 1 [file Table1.docx]

**Supplementary Table 1**: Embase search strategy using Ovid platform (January 1, 2013 to February 22, 2023)

| Search Number | Search Terms | Results |
| --- | --- | --- |
| 1 | ((immun* or autoimmun* or inflam*) adj3 resol*).mp. | 17164 |
| 2 | ((immun* or autoimmun* or inflam*) adj3 regulat*).mp. | 125375 |
| 3 | ((immun* or autoimmun* or inflam*) adj3 homeosta*).mp. | 17902 |
| 4 | (Resolution phase and (immun* or autoimmun* or inflam*)).mp. | 812 |
| 5 | ((immun* or autoimmun* or inflam*) adj3 rebalanc*).mp. | 153 |
| 6 | ((T cell adj regulat*) or Treg$).mp. | 53546 |
| 7 | ((IL-2 or IL2 or interleukin-2) adj3 mutein).mp. | 71 |
| 8 | ((pd 1 or pd1 or programmed cell death 1) adj3 agonist$).mp. | 147 |
| 9 | (CD200R$ adj3 agonist$).mp. | 43 |
| 10 | ((BTLA$ or lymphocyte attenuator) adj3 agonist$).mp. | 31 |
| 11 | immunometabolism.mp. | 2165 |
| 12 | immunoregulator$.mp. | 20379 |
| 13 | checkpoint inhibit$.mp. | 53943 |
| 14 | or/1-13 | 267050 |
| 15 | clinical trial/ | 1069243 |
| 16 | controlled clinical trial/ | 468642 |
| 17 | multicenter study/ | 368471 |
| 18 | Phase 3 clinical trial/ | 68010 |
| 19 | Phase 2 clinical trial/ | 105065 |
| 20 | randomized controlled trial$.mp. | 1097024 |
| 21 | Prospective Study/ | 856143 |
| 22 | retrospective study/ | 1424362 |
| 23 | cohort studies/ | 860368 |
| 24 | exp cross-sectional study/ | 545822 |
| 25 | (prospective or retrospective).ti. | 398494 |
| 26 | 15 or 16 or 17 or 18 or 19 or 20 or 21 or 22 or 23 or 24 or 25 | 4902020 |
| 27 | 14 and 26 | 32358 |
| 28 | rheumatoid arthritis/ | 209775 |
| 29 | exp asthma/ | 297907 |
| 30 | atopic dermatitis/ | 55354 |
| 31 | ulcerative colitis/ | 89455 |
| 32 | systemic lupus erythematosus/ | 108418 |
| 33 | or/28-32 | 718083 |
| 34 | 27 and 33 | 1990 |
| 35 | limit 34 to yr="2012 -Current" | 1440 |
| 36 | *consensus/ | 11988 |
| 37 | (expert opinion or consensus or working group).mp. | 374509 |
| 38 | 36 or 37 | 374509 |
| 39 | 14 and 33 and 38 | 164 |
| 40 | 35 or 39 | 1586 |
| 41 | limit 40 to yr="2013 -Current" | 1511 |

**Supplementary Table 2**: MEDLINE® search strategy using Ovid platform (January 1, 2013 to February 22, 2023)

| Search Number | Search Terms | Results |
| --- | --- | --- |
| 1 | ((immun* or autoimmun* or inflam*) adj3 resol*).mp. | 11787 |
| 2 | ((immun* or autoimmun* or inflam*) adj3 regulat*).mp. | 88483 |
| 3 | ((immun* or autoimmun* or inflam*) adj3 homeosta*).mp. | 13002 |
| 4 | (Resolution phase and (immun* or autoimmun* or inflam*)).mp. | 496 |
| 5 | ((immun* or autoimmun* or inflam*) adj3 rebalanc*).mp. | 107 |
| 6 | ((T cell adj regulat*) or Treg$).mp. | 29048 |
| 7 | ((IL-2 or IL2 or interleukin-2) adj3 mutein).mp. | 31 |
| 8 | ((pd 1 or pd1 or programmed cell death 1) adj3 agonist$).mp. | 57 |
| 9 | (CD200R$ adj3 agonist$).mp. | 31 |
| 10 | ((BTLA$ or lymphocyte attenuator) adj3 agonist$).mp. | 17 |
| 11 | immunometabolism.mp. | 1763 |
| 12 | immunoregulator$.mp. | 15536 |
| 13 | checkpoint inhibit$.mp. | 29217 |
| 14 | or/1-13 | 174645 |
| 15 | clinical trial.pt. | 537147 |
| 16 | controlled clinical trial/ | 95195 |
| 17 | multicenter study.pt. | 330907 |
| 18 | clinical trial, Phase III/ | 21410 |
| 19 | clinical trial, Phase II/ | 39298 |
| 20 | randomized controlled trial$.mp. | 845066 |
| 21 | Prospective Studies/ | 651608 |
| 22 | retrospective studies/ | 1096203 |
| 23 | cohort studies/ | 325145 |
| 24 | exp cross-sectional studies/ | 457211 |
| 25 | (prospective or retrospective).ti. | 274769 |
| 26 | 15 or 16 or 17 or 18 or 19 or 20 or 21 or 22 or 23 or 24 or 25 | 3446386 |
| 27 | 14 and 26 | 10643 |
| 28 | arthritis, rheumatoid/ | 108723 |
| 29 | exp asthma/ | 140698 |
| 30 | dermatitis, atopic/ | 24002 |
| 31 | colitis, ulcerative/ | 39977 |
| 32 | lupus erythematosus, systemic/ | 60560 |
| 33 | or/28-32 | 364837 |
| 34 | 27 and 33 | 424 |
| 35 | limit 34 to yr="2012 -Current" | 274 |
| 36 | *consensus/ | 3905 |
| 37 | (expert opinion or consensus or working group).mp. | 260523 |
| 38 | 36 or 37 | 260523 |
| 39 | 14 and 33 and 38 | 44 |
| 40 | 35 or 39 | 316 |
| 41 | limit 40 to yr="2013-Current" | 292 |

**Supplementary Table 3**: Trial characteristics and findings

| Author, year | Study design | Study or trial name/trial registration number | Treatment(s)  *Class* | Number of patients | Inclusion criteria | Exclusion criteria | Measures used to assess potential immune resolution | Clinical remission outcomes assessed for correlation with measures of potential immune resolution | Effect of intervention/conclusions |
| --- | --- | --- | --- | --- | --- | --- | --- | --- | --- |
| Atopic Dermatitis | | | | | | | | | |
| Weidinger, 2022(1) | Phase IIa randomized, double-blind placebo-controlled trial | NCT03754309 | Amlitelimab low dose, Amlitelimab high dose, and Placebo  *Anti-OX40 ligand (OX40L) monoclonal antibody* | 89 | Patients with moderate-to-severe AD | NR | Serum IL-22 levels | EASI score, SCORAD, and vIGA® | The decrease in IL-22 levels persisted for 36 weeks in patients defined as vIGA 0/1 responders at Week 16.  OX40L blockade is a promising treatment for AD that targets T-cell immune imbalance. |
| Guttman-Yassky, 2022(2) | Phase II randomized, double-blind placebo-controlled trial | NCT03703102 | Rocatinlimab  KHK4083  *Anti-OX40 monoclonal antibody* | For transcriptomic analysis: skin biopsy specimens from 20 patients;  for proteomic analysis: serum samples from 27 patients | Subjects with moderate-to-severe AD | NR | T helper (Th) 1/Th2/Th17/Th22, itch- (IL-22, IL-31), and skin-barrier–related gene expression | NR | Transcriptomic analysis revealed significant downregulation by OX40 blockade in Th2, Th1, Th17, and Th22 pathways, and in itch-related genes (i.e., IL-22, IL-31) and improved  skin-barrier–related gene (i.e. *FLG*) expression. |
| Guttman-Yassky, 2022(3) | Phase IIa randomized, double-blind placebo-controlled trial | JADE-MOA/NCT03915496 | Abrocitinib 200 mg, Abrocitinib 100 mg, Placebo  *JAK1-selective inhibitor* | 46 | Adults ≥18 years old with moderate-to-severe AD | NR | Th2 immune response (C-C motif chemokine ligand [*CCL*]*17*, *CCL18, CCL26*); Th17/Th22 immune response (S100 calcium-binding protein [*S100*]*A8, S100A9*, and *S100A12*) | IGA 0/1 response, and EASI-75 response | Compared with placebo, abrocitinib, a JAK1-selective inhibitor induced greater decreases of Th2 and Th17/Th22 immune response genes (*CCL17/18/26* and *S100A8/9/12*, respectively). |
| Rheumatoid Arthritis | | | | | | | | | |
| Wang, 2022(4) | RCT | ChiCTR-INR-16009546 | Conventional glucocorticoid and DMARD treatment, and conventional glucocorticoid and DMARD treatment + low-dose IL-2 | 41 patients with refractory RA and 40 healthy individuals | Patients between the ages of 18 and 65 had severely active rheumatoid arthritis [≥8 tender joints of 28  joints examined, ≥3 swollen joints of 28 joints examined, morning stiffness lasting longer than 60 min, a serum CRP level that is at least 1.5 times the upper limit or an ESR that is at least 28 mm per hour, and DAS28 ≥5.1]. At study entry, patients must have been regularly taking one or more conventional DMARDs for at least the preceding 6 months but without disease activity remission. Patients must also have a negative pregnancy test and agree to use effective contraception during the study and for at least 6 months after stopping study treatment. They were able to comply with scheduled visits, treatment plans, laboratory tests, and other study procedures. | Patients who had a history of malignancy, were suffering from the malignant disease within 5 years  prior to study entry, or had a recent clinically significant infection. Patients in a pregnancy test who disagreed with using effective contraception during the study or at least 6 months after stopping  study treatment. | CD4 Treg and Th17 counts, Th17/CD4 Treg ratio | DAS28 score, 28 tender joint count, 28 swollen joint count | Number of CD4 Treg was slightly negatively correlated with DAS28 (r = −0.625, p<0.001), ESR (r = −0.408, p=0.001), CRP (r = −0.344, p=0.009), number of joint pain (r = −0.639, p <0.001), and number of joint swollen (r = −0.538, p<0.001) while Th17/ CD4 Treg ratio was positively correlated with DAS28 (r = 0.350, p=0.004), number of joint pain (r =0.393, p=0.001), and number of joints swollen (r = 0.407, p=0.001) thus highlighting the relevance of deficiency of CD4 Treg and imbalanced Th17/CD4 Treg homeostasis in the disease activity.  Low-dose IL-2 decreased RA activity as assessed by DAS28, 28 tender joint count, and swollen joint count, and increased the number of CD4 Treg with a rebalancing of the Th17/CD4 Treg ratio. |
| Van Vollenhoven, 2018(5) | Phase IIb randomized, double-blind placebo-controlled trial | TREAT2b/NCT01999192 | Tregalizumab 25 mg, Tregalizumab 100 mg, Tregalizumab 200 mg    *Anti-CD4 monoclonal antibody* | 321 | Age ≥18 and ≤75 years.  Active rheumatoid arthritis according to the 1987 ACR or 2010 EULAR classification criteria with functional class I‐III for ≥6 months as defined by Hochberg et al. 1992.  Oral or parenteral methotrexate treatment for ≥12 weeks (overall), with an unchanged mode of administration and stable dose of ≥15 mg/week (or ≥12.5 mg/week in cases of methotrexate intolerance), but no more than the highest locally approved dose for rheumatoid arthritis, for ≥8 weeks prior to baseline. Methotrexate dose was expected to remain stable throughout the trial and could only be adjusted for safety reasons. If applicable, the dose of folic acid was to be unchanged for ≥8 weeks before baseline.* | Previous exposure to any systemic biologic therapy, JAK or SYK inhibitors, or tregalizumab. Previous anti‐TNF treatment was allowed if the treatment had been stopped at least 12 weeks or 5 half‐lives of the compound before baseline (whichever was longer) for other reasons than lack of efficacy or adverse events, and the treatment period did not exceed 6 weeks.  Treatment with conventional DMARDs apart from methotrexate in the 12 weeks before baseline, and for leflunomide in the 24 weeks before baseline (except where specific leflunomide wash‐out procedures were completed according to the guidelines).* | CD4 downmodulation | DAS remission in 28 joints (DAS28 <2.6); low disease activity by DAS 28 (DAS28 <3.2); Simplified Disease Activity Index (SDAI ≤11); Clinical Disease Activity Index (CDAI ≤10) | Although there was a clear dose-dependent decrease of CD4 expression by tregalizumab, there was no significant difference between tregalizumab and placebo regarding the number of patients with DAS remission (DAS28 <2.6). |
| Zhang, 2019(6)  Parent ref: Zhang, 2022(7) | Phase II randomized, double-blind placebo-controlled trial | NCT02467504 | Low-dose IL-2 with methotrexate, and placebo with methotrexate | 47 | NR | NR | Changes in Treg cell count, *CD56^bri^* NK cell count  Changes in *IL-17A*, *IFN-γ*, and IL-21 levels | DAS28-ESR remission | Low-dose IL-2 increased the number of Treg and *CD56^bri^* NK cells and decreased levels of IL-17A and IFN-γ, and decreased RA disease activity. Improvement by IL-2 therapy was correlated with higher serum levels of IL-21. |
| Zhang, 2022(7) | Phase II randomized, double-blind placebo-controlled trial | NCT02467504 | Low-dose IL-2 with methotrexate, and placebo with methotrexate | 47 | All patients (18–70 years) with RA fulfilled the revised 1987 ACR criteria, and were eligible if they had moderate to severely active disease, defined as DAS28-ESR >3.2. | Patients previously given biologic DMARDs or methotrexate; had an autoimmune disease other than RA, an active infection, recurrent bacterial infections, severe hepatic and renal dysfunction, or malignant tumor | The proportion of Treg cells, the proportion of Th17 cells | DAS28 remission (DAS28-ESR<2.6) | The low number of Treg and high IL-21 at baseline were associated with good clinical responses to low-dose IL-2 plus methotrexate. Also, compared with the reduction in Treg seen with methotrexate alone, low-dose IL-2 plus methotrexate increased the proportion of Treg. |
| Zhang, 2018(8)  Parent ref: Wen, 2019(9) | Double‐arm,  open‐label, phase1/2 trial | ChiCTR-IPR-17010307 | Sirolimus and conventional glucocorticoids and DMARDs, and conventional glucocorticoids and DMARDs  *mTOR inhibitor* | 52 analyzed | Eligible participants (aged 18 to 65 years) who fulfilled the revised 1987 ACR/EULAR criteria for the classification of RA and did not achieve remission with the conventional treatment over at least 6 months | The study excluded patients with allergy or intolerance to sirolimus, with malignant disease or a history of malignancy, or with chronic or severe infection | Treg and Th17 cells | DAS28 <2.6 | Sirolimus induced a higher rate of remission (DAS28<2.6) than conventional glucocorticoids and DMARDs and prevented the decrease in Treg and Th17 cells seen with conventional therapy alone. |
| Wang, 2019(10)  Parent ref: Wen, 2019(9) | Randomized, non-blinded, parallel-controlled trial | NR (ChiCTR-IPR-17010307) | Sirolimus and conventional treatment  *mTOR inhibitor* | 62 (55 analyzed) | NR | NR | Changes in levels of CD4+ T-cells subsets - Th1, Th2, Th17, and Treg cells | DAS28 score, ESR score, and number of tender joints and swollen joints | Sirolimus increased the number of Treg and reduced disease activity as assessed by DAS28, ESR, and the number of tender and swollen joints, with concomitant reduction in the use of immunosuppressive drugs for controlling disease, compared with conventional treatment. |
| Wen, 2019(9)  Related ref: Wang, 2018(11)  Niu, 2017(12) | Randomized, open-label, parallel-controlled trial | ChiCTR-IPR-17010307 | Sirolimus and conventional treatment  *mTOR inhibitor* | 62 (55 analyzed) | Patients fulfilled the 1987 and 2010 rheumatoid arthritis classification criteria, aged between 18 and 65 years, and had active disease (DAS28-ESR scores > 3.2) | Allergic or intolerant to sirolimus, suffering from malignant disease, had a history of malignancy, or had a recent clinically significant infection. | Levels of Th17 and Treg before and after therapy | Efficacy of sirolimus in reducing disease activity: DAS28-ESR score, ESR, tender joints count, and swollen joints count | Sirolimus decreased disease activity (DAS28, ESR, number of tender and swollen joints) and number of Th17 cells, and increased number of Treg. |
| Xu, 2015(13) | RCT | NR | Iguratimod, placebo  *Small-molecule DMARD* | 74 | All patients who fulfilled the 2010 diagnostic criteria of the ACR, had active disease and were consistent with the criteria of activity: ≥6 joints were swollen; ≥6 joints were tender; and fit two of the three criteria: (a) on the day of visiting, morning stiffness lasting more than 45 min; (b) ESR ≥28 mm/h; and (c) CRP ≥20 mg/dL | NR | Changes in frequencies of Th1, Th17, Tfh, Treg before and after therapy  Changes in the relative expression of Th1, Th17, Tfh, Treg, related transcriptional factors, and cytokines before and after therapy | DAS28 score | Iguratimod decreased disease activity (DAS28, ESR, CRP) and number of Th1 and Th17 cells, and increased number of Treg. |
| Li, 2017(14) | Prospective cohort | NR | Rapamycin plus low-dose IL-2 and methotrexate  *mTOR inhibitor* | 58 | DMARDs-naive RA patients | NR | Treg and Th17/Treg ratio | DAS28 score | Rapamycin plus low-dose IL-2 increased Tregs and decreased the Th17/Tregs ratio. |
| Bjarnadóttir, 2022(15) | Prospective cohort | NR | Infliximab  *Anti-TNFα* | 20 | Patients who fulfilled the ACR criteria for RA and were starting their first TNF inhibitor treatment | NR | Decrease in Th17/Tc17 ratio and increase in Tregs | DAS28-CRP ≤2.6 and CDAI ≤2.8 | Anti-TNFα treatment increased CD4+CD25+FoxP3+ Treg, and decreased Th17/Tc17 ratio at 6 months. |
| SLE | | | | | | | | | |
| Lai, 2022(16) | Prospective and RCT | Studies involved:   1. Prospective Study of Rapamycin for the treatment of SLE; NCT00779194 2. Treatment trial of SLE with  N-acetylcysteine; NCT00775476 | Sirolimus  *mTOR inhibitor* | 40 patients having active disease and 56 controls in prospective study;  73 patients with or without nephritis in a retrospective trial | Prospective study: (1) patients having active disease, (2) patients unresponsive or intolerant to conventional medications  Retrospective trial: patients with or without lupus nephritis | NR | Prospective study: Expansion of CD4+CD25+FoxP3+ Treg and CD8+ memory T cells, inhibition of IL-4 and IL-17 production by CD4+ and CD4-CD8- double-negative T-cells after 12 months  Retrospective trial: Increase in levels of C3 and C4 complement levels | NR; the study assessed BILAG, SLEDAI and prednisone use as measures of disease activity but did not refer to, or include specific definitions of, clinical remission. | Sirolimus increased levels of CD4+CD25+FoxP3+ Treg and CD8+ memory T-cells and reduced IL-4 and IL-17 production by CD4 T-cells and CD4- CD8- double-negative T-cells in patients with SLE. It also increased C3 and C4 complement levels, and reduced steroid use in patients with or without SLE-associated renal disease. |
| Shipa, 2022(17) | RCT | BEAT-lupus trial | Belimumab and rituximab, and placebo and rituximab  *Monoclonal antibody that inhibits B-cell activating factor (BAFF)* | 52 | NR | NR | Serum autoantibodies (IgA anti-dsDNA) and cytokines, peripheral blood RNA expression, B-cell flow cytometry | Major clinical response (defined as the reduction to BILAG C in all domains, steroid dose of ≤7.5 mg/day & SLEDAI≤2, without anti-dsDNA antibody component) at 52 weeks | Belimumab after rituximab not only increased the number of anergic/resting B cells (unswitched memory B cells, p=0.045, and anergic naïve B cells, p=0.046) and reduced potentially pathogenic B-cell subsets (activated naïve, activated switched memory and double-negative memory B-cell, p< 0.001, 0.032, 0.021 respectively) but also targets IgA2 anti-dsDNA antibody production that could be used as a baseline predictive biomarker of clinical response to belimumab following rituximab combination therapy. |
| Chu, 2019(18) | RCT | NR | Rapamycin, and Rapamycin plus all-trans retinoic acid  *mTOR inhibitor* | 70 | All patients conformed to the ACR 1997 revised criteria for the classification of SLE | NR | Th17 cell count, Treg cell count, Th17/Treg ratio | NR; the study assessed change in glucocorticoid requirement and in SLEDAI score as measures of treatment efficacy but did not directly refer to, or include any specific definitions for low disease activity or clinical remission | Disease activity in SLE was reduced with rapamycin used either alone or in combination with all-trans retinoic acid, with an accompanying reduction in glucocorticoid requirement. There was a decrease of Th17 cells and of the ratio Th17/Treg, and an increase in the number of Treg but no difference between the two treatment groups. The drug’s beneficial effect may relate to the regulation of the Th17/Treg cell balance. |
| Shao, 2019(19) | Randomized, double-blind, placebo-controlled clinical trial | NR | IL-2 plus standard care, and placebo plus standard care | 60 | NR | NR | % of patients with complete remission of LN,  Treg | SELENA-SLEDAI score, SLE Responder Index-4 (SRI-4), glucocorticoid requirement | Low-dose IL-2 increased the number of Treg and NK cells, and induced remission, and reduced glucocorticoid requirement, in active SLE. |
| Jing, 2017(20) | Prospective cohort | NR | Low dose IL-2 combined with rapamycin  *mTOR inhibitor* | 82 | Patients in line with the standard of ACR in 1997, who are treated with glucocorticoid and immunosuppressant for more than one year, "but the subjects continue to rise to a peak". | NR | Treg, Th 17 cells, Th17/Treg ratio | NR | Low-dose IL-2 plus rapamycin increased the number of Treg and decreased the ratio of Th17/Treg without a change on the number of Th17 cells. |
| Lipsky, 2022(21) | Phase IIb, randomized, double-blind, placebo-controlled trial | NCT03161483 | Iberdomide 0.15 mg, Iberdomide 0.3 mg, Iberdomide 0.45 mg and placebo  *Cereblon-modifying (CM) agent* | 288 | Adults (≥18 years of age) with a diagnosis of SLE for at least 6 months, a SLEDAI 2000 score ≥6 points and positive for autoantibodies associated with SLE | Active, severe, or unstable neuropsychiatric lupus disease, antiphospholipid syndrome or history of thrombosis, estimated glomerular filtration rate <45 mL/min/1.7 m^2^ or proteinuria >2000 mg/d, or active lupus nephritis, which may require induction therapy | Cell count of whole blood leucocytes including Treg, Th17, and Tfh cells,  Plasma cytokines including IL-2 | NR; the study assessed SRI-4 at Week 24 as measure of treatment efficacy but did not directly refer to, or include any specific definitions for low disease activity or clinical remission | Iberdomide reduced activity of the B-cell pathway, and increased Treg and IL-2 levels, this suggesting rebalancing of immune abnormalities associated with SLE. |
| Miao, 2021(22) | Post hoc analysis from a double-blind  RCT | NCT02465580 | Low-dose IL-2, and placebo | 60 | Active SLE patients who had an inadequate response to standard treatment for ≥3 months | NR | Proportion and absolute number of Treg, CXCR5+PD-1lowTreg, CXCR5+PD-1highTreg, Tfh, Tfh1, Tfh2, Tfh17. Ratios of Treg/Tfh, CXCR5+PD-1lowTreg/Tfh, CXCR5+PD-1highTreg/Tfh, Treg/Tfh17, CXCR5+PD-1lowTreg/Tfh17, CXCR5+PD-1highTreg/Tfh17 | Imbalanced Tfh and Tfr cell association with disease activity (SLEDAI score) | A Tfr/Tfh cell imbalance at baseline was associated with increased disease activity as assessed by the SLEDAI score (r=0.273, p=0.052). The clinical benefit of low-dose IL-2 is associated with restoration of Tfr/Tfh cell balance. |
| Humrich, 2022(23)  Humrich, 2022(24) | Phase II, randomized, double-blind, placebo-controlled trial | LUPIL-2/NCT02955615 | Low-dose IL-2 (ILT-101), and placebo | 100 | Eligible patients were aged 18 years or older with a confirmed diagnosis of SLE according to the revised classification criteria of the ACR from 1997 or of the Systemic Lupus International Collaborating Clinics and having a moderate-to-severe disease activity characterized by a SELENA-SLEDAI score of at least 6 at baseline and the presence of antinuclear antibodies with a titer of ≥1:160 Patients had to be under stable background therapy.* | Serious organ failure. Any clinical evidence of active chronic infection HIV, hepatitis B, hepatitis C. Clinically significant pleuritis or pericarditis. Type 1 diabetes and/or Crohn's disease.  Use of Benlysta (belimumab) in the past 4 weeks. Use of rituximab in the past 6 months. Vaccination with live attenuated virus in the last month.* | The numbers and percentages of CD3+CD4+FoxP3+CD127loCD25hi Treg subset (CD25hi Treg), and other immune cells. | Clinical remission: SELENA-SLEDAI ≤2  Reduction in prednisone daily dose | Low-dose IL-2 increased the proportion of patients in clinical remission (SELENA-SLEDAI ≤2) and the number and proportion of Treg. Only patients experiencing a clinical response to IL-2 had significant increases in Treg levels, so suggesting that clinical outcome is related to the scale of the Treg response. |
| Fanton, 2022(25) | Phase I, randomized, double-blind, placebo-controlled trials | SAD, NCT04133116 and MAD, NCT03556007 | NKTR-358 (polyethylene glycol [PEG]-IL-2 conjugate), and placebo | SAD: 100, MAD: 48 | The SAD study enrolled healthy adults aged 18 to 55 years. In the MAD study, eligible patients had been diagnosed with SLE for at least 6 months, meeting at least four of the 11 American College of Rheumatology criteria for SLE (at least one of which must have been: positive antinuclear antibody test titer of ≥1:80 at screening; above normal anti-double-stranded DNA antibodies at screening; or above normal anti-Smith antibody at screening). Patients were required to have minimal-to-moderate SLE disease activity, and to be on a stable dose of concomitant medications for ≥8 weeks before the study start. | SAD: Had previous or concurrent immune-mediated disease, other relevant medical conditions, or were taking confounding medications. MAD: Patients with active lupus nephritis or central nervous system disease | Total CD4+ Treg and CD25^bright^ Treg | Disease activity was assessed as an exploratory end point in the MAD study, as measured by the SLE Disease Activity Index (SLEDAI), Cutaneous Lupus Erythematosus Disease Area and Severity Index–Activity (CLASI-A), and joint counts | A polyethylene glycol (PEG)-IL-2 conjugate increased the number and proportion of CD4+ Treg and CD25^bright^ Treg in a dose-dependent manner. |
| Ulcerative Colitis | | | | | | | | | |
| Schreiber, 2021(26) | Open-label, prospective phase IIa trial | EudraCT no., Nu 2016-000205-36 | Olamkicept  *Gp130Fc-fusion-protein, anti-IL-6* | 16 (9 with UC, 7 with CD) | Patients (aged 21–66 years) with moderately to severely active UC and CD; experienced failure with conventional therapies with no more than 2 prior biologics (limited to anti-TNFs and/or vedolizumab) | NR | The change of a mucosal proinflammatory gene signature (*TNF, IL1A, REG1A, IL8, IL1B,* and *LILRA*) as a composite score from baseline to Week 14. The composite score consists of a set of genes that represent the level of mucosal inflammation in mucosal tissue. The primary assessment was remission. | Clinical remission as defined by a Mayo score of ≤2, rectal bleeding score of 0, and endoscopy of ≤1 for UC | Olamkicept, an inhibitor of IL-6, induced clinical remission in around 20% of patients with UC, and a specific gene signature (involving reduced proinflammatory gene expression) in UC. |

*A full list of inclusion and exclusion criteria are reported in the supplementary material for the published paper.

ACR, American College of Rheumatology; AD, atopic dermatitis; CD, Crohn’s disease; CRP, C-reactive protein; DAS28, Disease Activity Score-28 joints; DAS28-ESR, Disease Activity Score-28 joints‐erythrocyte sedimentation rate; DMARDs, disease-modifying antirheumatic drugs; EASI, Eczema Area and Severity Index ; ESR, erythrocyte sedimentation rate; EULAR, European Alliance of Associations for Rheumatology; IFN, interferon; IL, interleukin; IL1A, interleukin 1 alpha; IL1B, interleukin 1 beta; JAK, Janus kinase; LILRA, leukocyte immunoglobulin-like receptors A; MAD, multiple ascending dose; mTOR, mammalian target of rapamycin; NA, not applicable; NK, natural killer; NR, not reported; RCT, randomized controlled trial; REG1A, regenerating family member 1 alpha; RNA, ribonucleic acid; SAD; single ascending dose; SCORAD, SCORing Atopic Dermatitis; SELENA-SLEDAI, Safety of Estrogens in Lupus Erythematosus National Assessment-Systemic Lupus Erythematosus Disease Activity Index; SLE, systemic lupus erythematosus; SYK, spleen tyrosine kinase; Tfh, T follicular helper cells; Tfr, T follicular regulatory cells; TNF, tumor necrosis factor; Treg, regulatory T-cells; UC, ulcerative colitis; UK, United Kingdom; US, United States; vIGA, validated Investigator Global Assessment.

**References**

1. Weidinger S, Cork M, Reich A, Bieber T, Gilbert S, Brennan N, et al. Treatment with Amlitelimab - a Novel Non-Depleting, Non-Cytotoxic Antiox40ligand Monoclonal Antibody - Reduces IL-22 Serum Levels in a Phase 2a Randomized, Placebo-Controlled Trial in Patients with Moderate-to-Severe Atopic Dermatitis. *31st EADV Congress*; September 7-10; Milan, Italy(2022). https://eadv.org/scientific/abstract-books/. Accessed September 21, 2023.

2. Guttman-Yassky E, Kishi A, Kashima N, Brandusa-Pavel A, Shimabe M, Ohashi R, et al. KHK4083/AMG 451 (Rocatinlimab), an Anti-OX40 Monoclonal Antibody, Provides Durable Transcriptomic Improvement in Skin with Atopic Dermatitis Patients. *31st EADV Congress*; September 7-10; Milan, Italy(2022). https://eadv.org/scientific/abstract-books/. Accessed September 21, 2023.

3. Guttman-Yassky E, Weidinger S, Bissonnett R, Armstrong AW, Seneschal J, Eyerich K, et al. Abrocitinib Reduces the Levels of Key Biomarkers Associated with Atopic Dermatitis in the Skin of Patients with Moderate-to-Severe Atopic Dermatitis. *31st EADV Congress*; September 7-10; Milan, Italy(2022). https://eadv.org/scientific/abstract-books/. Accessed September 21, 2023.

4. Wang J, Zhang SX, Chang JS, Cheng T, Jiang XJ, Su QY, et al. Low-Dose IL-2 Improved Clinical Symptoms by Restoring Reduced Regulatory T Cells in Patients with Refractory Rheumatoid Arthritis: A Randomized Controlled Trial. *Front Immunol* (2022) 13:947341. Epub 2022/12/17. doi: 10.3389/fimmu.2022.947341.

5. van Vollenhoven RF, Keystone EC, Strand V, Pacheco-Tena C, Vencovsky J, Behrens F, et al. Efficacy and Safety of Tregalizumab in Patients with Rheumatoid Arthritis and an Inadequate Response to Methotrexate: Results of a Phase IIb, Randomised, Placebo-Controlled Trial. *Ann Rheum Dis* (2018) 77(4):495-9. Epub 2018/01/19. doi: 10.1136/annrheumdis-2017-212478.

6. Zhang X, He J, Zhang R, Liu X, Chen J, Sun X, et al. Efficacy and Safety of Low-Dose Interleukin-2 in Combination with Methotrexate in Patients with Active Rheumatoid Arthritis: Clinical Results from the Randomized, Double-Blind, Placebo-Controlled Trial. *Eur J Immunol* (2019) (Supplement 3):1852-3. doi: 10.1002/eji.201970400.

7. Zhang X, Miao M, Zhang R, Liu X, Zhao X, Shao M, et al. Efficacy and Safety of Low-Dose Interleukin-2 in Combination with Methotrexate in Patients with Active Rheumatoid Arthritis: A Randomized, Double-Blind, Placebo-Controlled Phase 2 Trial. *Signal Transduct Target Ther* (2022) 7(1):67. Epub 2022/03/08. doi: 10.1038/s41392-022-00887-2.

8. Zhang SX, Wang J, Yang WX, Yu NN, Wen HY, Gao C, et al. Efficacy and Safety of Sirolimus Treatment in Patients with Rheumatoid Arthritis: A Double-Arm, Open-Label, Phase1/2 Trail. *Int J Rheumatic Dis* (2018) 21(Supplement 1):228-9. doi: 10.1111/1756-185X.13361.

9. Wen HY, Wang J, Zhang SX, Luo J, Zhao XC, Zhang C, et al. Low-Dose Sirolimus Immunoregulation Therapy in Patients with Active Rheumatoid Arthritis: A 24-Week Follow-up of the Randomized, Open-Label, Parallel-Controlled Trial. *J Immunol Res* (2019) 2019:7684352. Epub 2019/11/30. doi: 10.1155/2019/7684352.

10. Wang J, Zhang SX, Hu FY, Zheng XJ, Cheng T, Yu NN, et al. The Efficacy and Safety of Sirolimus in Patients with Active Rheumatoid Arthritis: A Randomized and Parallel-Controlled Clinical Trial. *Ann Rheumatic Dis.* (2019) 78(Supplement 2):738. doi: 10.1136/

annrheumdis-2019-eular.3906

11. Wang J, Zhang Sx, Hu Fy, Zheng Xj, Cheng T, Yu Nn, Et Al. Sirolimus Treatment in Patients with Refractory Rheumatoid Arthritis: A Double-Arm, Open-Label, Phase1/2 Trail [Abstract]. Arthritis Rheumatol. 2018; 70 (Suppl 9). https://acrabstracts.org/abstract/sirolimus-treatment-in-patients-with-refractory-rheumatoid-arthritis-a-double-arm-open-label-phase1-2-trail/. Accessed September 21, 2023.

12. Niu H, Li Z, He J, Jia R, Luo J, Gao C, et al. Ab0421 Rapamycin Reduces Disease Activity through Restoring Regulatory T Cell Numbers in Patients with Active Refractory Rheumatoid Arthritis. *Ann Rheumatic Dis.* (2017) 76(Suppl 2):1197-. doi: 10.1136/annrheumdis-2017-eular.2701.

13. Xu Y, Zhu Q, Song J, Liu H, Miao Y, Yang F, et al. Regulatory Effect of Iguratimod on the Balance of Th Subsets and Inhibition of Inflammatory Cytokines in Patients with Rheumatoid Arthritis. *Mediators Inflamm* (2015) 2015:356040. Epub 2015/12/30. doi: 10.1155/2015/356040.

14. Li Z, Niu H, Chen M, Yao H, Luo J, Gao C, et al. Efficiency and Safety of Rapamycin Combined with Low-Dose IL-2 Treatment Compared with Methotrexate in Patients with Rheumatoid Arthritis. *Ann Rheumatic Dis.* (2017) 76(Supplement 2):268. doi: 10.1136/annrheumdis-2017-eular.4575

15. Bjarnadottir U, Einarsdottir HK, Stefansdottir E, Helgason EA, Jonasdottir D, Gudmundsson S, et al. Resolution of Th/Tc17-Driven Inflammation During Anti-TNFα Treatment of Rheumatoid Arthritis Reveals a Unique Immune Biomarker Profiling Pattern. *Scand J Immunol* (2022) 95(1):e13116. Epub 2022/02/26. doi: 10.1111/sji.13116.

16. Lai ZW, Kelly R, Winans T, Marchena I, Shadakshari A, Yu J, et al. Blockade of the Mechanistic Target of Rapamycin Elicits Rapid and Lasting Improvement of Disease Activity through Restraining Pro-Inflammatory T Cell Lineage Specification in Patients with Active SLE. *Lupus Sci Med* (2022) 9(Supplement 3):A86-A8. doi: 10.1136/lupus-2022-lupus21century.85

17. Shipa M, Santos LR, Nguyen DX, Embleton-Thirsk A, Parvaz M, Isenberg D, et al. Belimumab after Rituximab Targets IgA2 Anti-dsDNA Antibody Production and Shifts Repopulating B-Cells Towards an Anergic, Nonpathogenic Phenotype in Systemic Lupus Erythematosus. *Arthritis Rheumatol.* (2022) 74(Supplement 9):1930-2. doi: 10.1002/art.42355

18. Chu Y, Zhao C, Zhang B, Wang X, Wang Y, An J, et al. Restoring T-Helper 17 Cell/Regulatory T-Cell Balance and Decreasing Disease Activity by Rapamycin and All-Trans Retinoic Acid in Patients with Systemic Lupus Erythematosus. *Lupus* (2019) 28(12):1397-406. Epub 2019/09/26. doi: 10.1177/0961203319877239.

19. Shao M, He J, Zhang R, Zhang X, Chen J, Sun X, et al. Low-Dose Interleukin-2 Treatment of Active SLE with No Increase in Infection Risk. *Eur J Immunol* (2019) 49(Supplement 3):972. doi: 10.1002/eji.201970400

20. Jing X, Liu X, Wang J, Qiao Y, Liang Z, Hao M, et al. Refractory Systemic Lupus Erythematosus Is Mainly Associated with the Decreased Number of Regulatory T Cells and Low-Dose IL-2 Combined with Rapamycin Can Efficiently Recovery the Balance of Th17/Regulatory T Cells. *Ann Rheumatic Dis.* (2017) 76(Supplement 2):872-3. doi: 10.1136/annrheumdis-2017-eular.4552

21. Lipsky PE, Vollenhoven RV, Dörner T, Werth VP, Merrill JT, Furie R, et al. Biological Impact of Iberdomide in Patients with Active Systemic Lupus Erythematosus. *Ann Rheum Dis* (2022) 81(8):1136-42. Epub 2022/04/29. doi: 10.1136/annrheumdis-2022-222212.

22. Miao M, Xiao X, Tian J, Zhufeng Y, Feng R, Zhang R, et al. Therapeutic Potential of Targeting Tfr/Tfh Cell Balance by Low-Dose-Il-2 in Active SLE: A Post Hoc Analysis from a Double-Blind Rct Study. *Arthritis Res Ther* (2021) 23(1):167. Epub 2021/06/13. doi: 10.1186/s13075-021-02535-6.

23. Humrich JY, Cacoub P, Rosenzwajg M, Pitoiset F, Pham HP, Guidoux J, et al. Low-Dose Interleukin-2 Therapy in Active Systemic Lupus Erythematosus (Lupil-2): A Multicentre, Double-Blind, Randomised and Placebo-Controlled Phase II Trial. *Ann Rheum Dis* (2022) 81(12):1685-94. Epub 2022/08/17. doi: 10.1136/ard-2022-222501.

24. Humrich J, Cacoub P, Rosenzwajg M, Pitoiset F, Pham H, Guidoux J, et al. S11.3 Low-Dose Interleukin-2 Therapy in Active Systemic Lupus Erythematosus (LUPIL-2): A Multi-Center, Double-Blind, Randomized and Placebo-Controlled Phase 2 Trial. *Lupus Sci Med* (2022) 9(Suppl 2):A16-A7. doi: 10.1136/lupus-2022-elm2022.24.

25. Fanton C, Furie R, Chindalore V, Levin R, Diab I, Dixit N, et al. Selective Expansion of Regulatory T Cells by NKTR-358 in Healthy Volunteers and Patients with Systemic Lupus Erythematosus. *J Transl Autoimmun* (2022) 5:100152. Epub 2022/05/07. doi: 10.1016/j.jtauto.2022.100152.

26. Schreiber S, Aden K, Bernardes JP, Conrad C, Tran F, Hoper H, et al. Therapeutic Interleukin-6 Trans-Signaling Inhibition by Olamkicept (sgp130Fc) in Patients with Active Inflammatory Bowel Disease. *Gastroenterology* (2021) 160(7):2354-66 e11. Epub 2021/03/06. doi: 10.1053/j.gastro.2021.02.062.
